# Root canal configuration and root wall thickness of first maxillary premolars in an Israeli population. A Cone-beam computed tomography study

**DOI:** 10.1038/s41598-019-56957-z

**Published:** 2020-01-16

**Authors:** Anda Kfir, Olga Mostinsky, Orly Elyzur, Moran Hertzeanu, Zvi Metzger, Ajinkya M. Pawar

**Affiliations:** 1 Department of Endodontology, Tel Aviv University, Tel Aviv, Israel; 2 Department of Conservative Dentistry and Endodontics, Nair Hospital Dental College and Hospital, Mumbai, Maharashtra India

**Keywords:** Oral anatomy, Dental pulp

## Abstract

Anatomical features of first maxillary premolars may greatly affect endodontic and following restorative treatments. The aim of this study was to evaluate root canal configuration and root wall thickness of first maxillary premolars using a preexisting CBCT database. A CBCT database of 400 first maxillary premolar was used to study canal configuration, presence of furcation-facing groove on the buccal root and root wall thickness. Root wall thickness was measured from axial CBCT slices at three critical points of the root: The most coronal part of the furcation-facing groove in the buccal root, when present, the CEJ level of the palatal root and 5 mm apically to the CEJ level of the palatal root. Vertucci Type IV configuration was the most common among all teeth, but in single-rooted teeth, Vertucci Type II was predominant. The mean thickness of the buccal root in the area of a furcation-facing groove was 1.1 (±0.2) mm, but in 39% of the cases, it was thinner than 1 mm. The mean thickness of the palatal root at 5 mm from the CEJ was 1.1 (±0.2), but in 28% of the cases, it was thinner than 1 mm. Thickness of root dentin walls of first maxillary premolars varies and may be limited at critical points in both buccal and palatal roots. In case the patient has a previous CBCT scan it may be useful for planning treatment of first maxillary premolars, in order to recognize and avoid potential risks such as furcation-facing groove, thin dentin walls in critical areas and presence of Type II Verucci canal, all of which may dictate less invasive procedures, using smaller files.

## Introduction

Endodontic treatment requires knowledge of and familiarity with root anatomy, root canal morphology and their commonly occurring variations. Such variations may include the number of roots, the number and configuration of the root canals and the frequency of their occurrence^1–3^.

Cone-beam computed tomography is an accepted method to study and visualize the ambiguous morphology of an individual tooth^4^. Traditional radiographic methods present the anatomy of the root as a two-dimensional plenary projection. On the other hand, CBCT provides three-dimensional imaging that offers the possibility to view an individual tooth in any plane, rather than only in the predetermined “default views” of periapical radiographs^4^.

Recently, the AAE and AAOMR Joint Position Committee (2015) indicate in its 3^rd^ recommendation that “Limited FOV CBCT should be considered the imaging *modality of choice* for initial treatment of teeth with the potential for extra canals and suspected complex morphology, such as mandibular anterior teeth, and maxillary and mandibular *premolars* and molars”^5^. However, the same committee also clearly stated in its Recommendation #1 that “Intraoral radiographs should be considered the imaging modality of choice in the evaluation of the endodontic patient”^5^. Thus, CBCT should *not* be used for routine dental screening.

Nevertheless, large databases of CBCT images, which were previously acquired for a variety of clinical reasons, may provide valuable information about the typical root morphology for a given population. Furthermore, such existing CBCT volumes may allow measurements and thus quantitative analysis of the dimensions of the roots and root canals and more specifically the *thickness* of the root walls^6^. Measurements obtained from CBCT images showed concordance with the gold standard (cadaver specimen measurements) and are considered an acceptable method for estimating the dimensions of different anatomical landmarks^4,7^.

First maxillary premolars often present a variable and complex anatomy. One of the important anatomical features of the first maxillary premolar is the potential presence of a furcation groove in the palatal aspect of the buccal root. The prevalence of such a furcation groove was reported to be 62–100%^8^. The thickness of the dentin wall at such grooves, in natural, un-instrumented root canals, has been reported to be less than one millimeter^9^. Recognition of such an anatomical feature, which cannot be seen in a periapical radiograph, is important to prevent excessive thinning and even strip-perforation of the dentin wall in this area. Data on the canal diameter and root wall thickness at the area of such groove are of great importance and may help in estimating the risk of procedural errors in the buccal root of first maxillary premolars.

When a post and core are required in the first maxillary premolar, the post is commonly placed in the palatal root. Therefore, the diameter of the canal and the root wall thickness at the area that is expected to be prepared for the post may also be of particular interest and importance. Several studies have shown that posts must be surrounded by at least 1 mm thickness of sound dentin, if the long-term integrity of the root is to be preserved^3,10^.

It should be noted that endodontically treated and restored maxillary first premolars are among the teeth that are most prone to vertical root fractures^11^. This tendency may be related to the unique anatomical features of this tooth and, especially, to excessive thinning of the root walls by the endodontic and subsequent restorative procedures.

A recent review^3^ about the anatomy and morphology of the maxillary first premolars included four studies performed using CBCT; however, none of these studies addressed the issue of dentin thickness of the roots prior to instrumentation^12–14^.

The aim of the present study was to investigate several parameters of the anatomy of maxillary first premolars, using an existing CBCT database. The parameters studied were the number of roots, the number and configuration of the root canals, and the prevalence of the furcation-facing (palatal) groove in the buccal root. Additionally the present study emphasized yet another aspect of variability: root wall thickness of first maxillary premolars that also presents with high variability. The root wall thickness was measured at (i) the most coronal part of the palatal groove of the buccal root (if present) and (ii) the palatal root at the CEJ and at a level of 5 mm apical to the CEJ, where a post preparation will commonly reach.

## Materials and Methods

CBCT scans of 400 first maxillary premolars were obtained from the dental school’s CBCT database (The Goldschleger School of Dental Medicine, Tel Aviv University, Tel Aviv, Israel). All patients were initially referred to CBCT as a part of their dental examination, diagnosis and treatment plan; the reason for all referrals was not related to the current study. The study was approved by the Tel Aviv University Institutional Ethics Committee (Certificate 132.16).

The study included only fully developed maxillary first premolars with intact roots. Previously treated teeth and teeth with open apices were excluded. Teeth with resorption were also excluded from the present study. All CBCT images were previously produced using a Planmeca ProMax 3d classic CBCT scanner (Planmeca, Helsinki, Finland), which was operated at 90 kV and 14 mA, with a field of view (FOV) of 8 × 5 cm. The voxel size of the scans was 150 µm. The slice thickness was 0.150 mm. The CBCT scans were analyzed using Planmeca Romexis software (Planmeca, Helsinki, Finland) in a partially darkened room.

The 400 teeth were selected from CBCT scans performed during 2016–2018. This selection consisted of checking sequential scans and selecting those who had at least one first maxillary premolar that fitted the inclusion criteria above, one tooth per patient. Sixty seven percent of the 400 patients were female while 33% were male, with age ranged from 20 to 73 with a mean of 41 Y.

**Figure 1:**
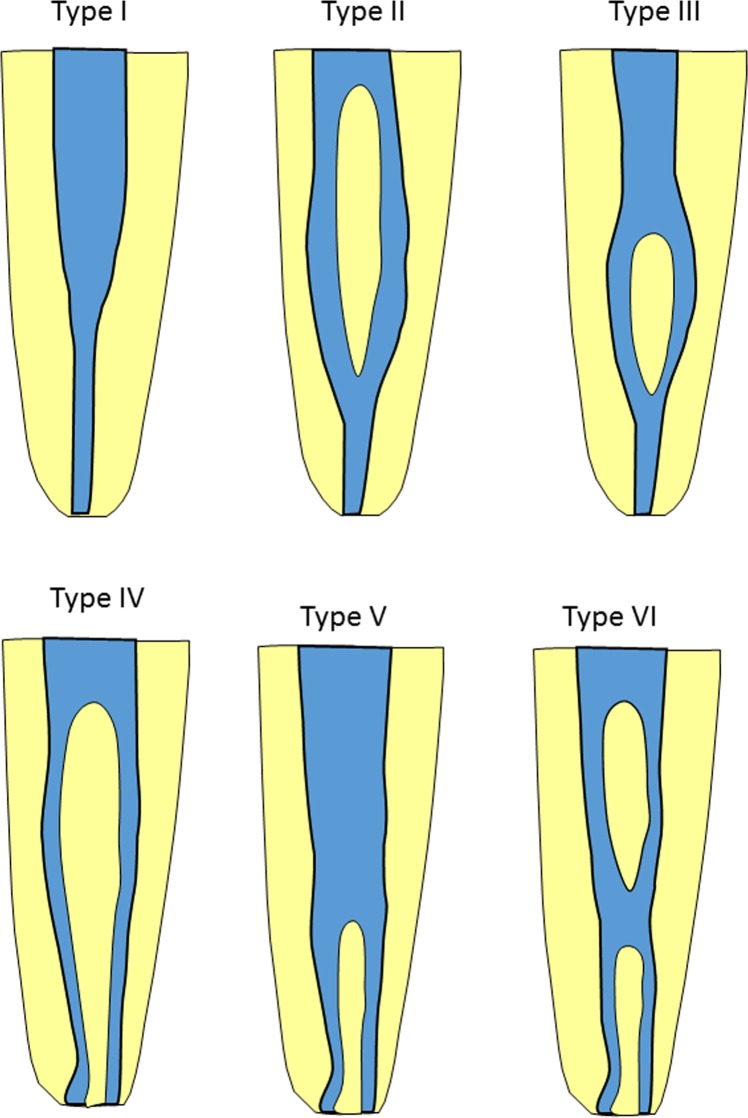
Schematic presentation of Vertucci Type I-VI root canal configuration. For simplicity, the canal configuration is presented on the image of a single rooted premolar root.

All teeth were analyzed in 3 planes: the sagittal, coronal and axial planes to determine the number of roots and root canal configuration. The configuration of the root canals was defined according to the Vertucci Type system (Fig. 1), in which Vertucci Type I is single canal, which runs from orifice to apex, Vertucci Type ll is two canals arise from pulp chamber and during their course unite into one. Vertucci type lll is one canal arises from pulp chamber and during its course splits into two. These two canals again unite into one before exiting from apex. Vertucci Type lV is two canals that run separately from orifice to apex. Vertucci Type V is one canal which arises from floor of pulp chamber, and during its course divides into two, and Vertucci Type Vl is two canals start from pulp chamber, during their course; they unite into one and then again divide into two before exiting from root apex^15^. A tooth was considered a two-rooted tooth when it had two distinct roots, either diverging or fused, with two canals and two apices^16^.

The analysis of each tooth was performed independently by two senior postgraduate endodontic students (O.M. and O.D.E.) and repeated after 3 week interval. The readings of the two observers were compared, and in an event of disagreement, the case was discussed until a consensus was reached. The Kappa value for the intra-observer agreement was 0.85 for both observers and 0.80 for the inter-observer agreement.

To obtain axial sections for wall thickness measurements, the image of the tooth to be analyzed was first rotated, so that the sagittal and coronal planes aligned, then axial plane images(“slices”), perpendicular to the long axis of the root, were obtained at the desired levels (see below) and these images were recorded and stored for future measurements.

The thickness of the root canal walls was later measured from these axial plane images which were taken from three locations: (i) the most coronal part of the furcation groove, when present in the buccal root; (ii) the palatal root at the CEJ level; and (iii) the palatal root at 5 mm apical to the CEJ. All measurements were performed using the measurement tools of the Planmeca software. A line was drawn on the image, representing the diameter of the root at the desired area, and measurements of the wall thickness were done along this line by two observers who were first calibrated for performing such measurements. Each observer performed the measurements independently and repeated the measurements after a two weeks interval. The average of these 4 repeated measurements was recorded as the study parameter.

The data are presented as the mean and as the median value with the range.

### Ethical approval

All procedures performed in studies involving human participants were in accordance with the ethical standards of the institutional and/or national research committee and with the 1964 Helsinki declaration and its later amendments or comparable ethical standards. The study was also approved by the Tel Aviv University Ethics Comity (Certificate 132.16).

### Informed consent

Informed consent was obtained from all individual participants included in the study.

## Results

### Number of roots and root canals

A total of 400 maxillary first premolars were studied using CBCT scans. Among these teeth, a single root was found in 36% (143/400) of the cases, two roots were found in 61% (245/400) of the cases, and 3 roots were found in 3% (12/400) of the cases.

The vast majority of the teeth had 2 canals (95%) (380/400), followed by 3 canals (3%) (12/400) and one canal (2%) (8/400).

### Configuration of canal system

The most prevalent configuration of root canal system in the whole study population was Vertucci Type IV: 74% (295/400). Other canal system configurations were found in smaller numbers (Table 1).

**Table 1:** Root canal configuration in first maxillary premolars.

	Vertucci Type I	Vertucci Type II	Vertucci Type III	Vertucci Type IV	Vertucci Type V	Vertucci Type VI
Total premolar sample (n = 400)	2% (7/400)	17% (67/400)	0.5% (2/400)	74% (295/400)	0.5% (2/400)	6% (27/400)
Single rooted premolars (n = 143)	5% (7/143)	48% (69/143)	3% (4/143)	31% (45/143)	1% (1/143)	12% (17/143)

Evaluated from CBCT scans.

In the single-rooted first maxillary premolars, Vertucci Type II was the most prevalent root canal configuration (48%) (69/143), followed by Vertucci Type IV (31%) (45/143). Other Vertucci types were found in smaller numbers (Table 1). The incidence of the various Vertucci Types in single rooted first maxillary premolars was significantly different from that of the total study population (p < 0.01). In the above calculations the tooth with either one or two roots, was considered as a unit.

A furcation groove in the palatal wall of the buccal root was found in 58% (142/246) of two-rooted teeth.

### Wall thickness measurements - buccal root

When the furcation groove was present on the palatal side of the buccal root, the mean dentin thickness at the most coronal part of the furcation groove was 1.1 (±0.2), with a median of 1.1 and a range of 0.6 to 1.8 mm (Table 2). In 39% of the cases, the thickness was below 1.0 mm, and in 11% of the cases, it was thinner than 0.8 mm (Table 2).

**Table 2:** Thickness of the dentin canal wall at various locations in first maxillary premolars*.

Location	Wall Thickness Mean (±SEM) mm	Wall Thickness Median mm	Wall Thickness Range mm	Wall Thickness < 1.0 mm Percent of Cases	Wall Thickness < 0.8 mm Percent of Cases
Groove in the palatal aspect of the buccal root**	1.1 (±0.2)	1.1	0.6–1.8	39%	11%
Palatal root at CEJ level	2.3 (±0.4)	2.4	1.0–3.3		
Palatal root at 5 mm apical to CEJ	1.1 (±0.2)	1.1	0.6–1.5	28%	3%

*Measured from axial CBCT slices.

**When such groove present.

### Wall thickness measurements - palatal root

The mean thickness of the canal wall of the palatal root at the level of the CEJ was 2.3 (±0.4) mm (n = 189), with a median of 2.4 mm and a range of 1.0 mm to 3.3 mm (Table 2).

The mean thickness of the palatal canal wall at 5 mm apical to the CEJ was 1.1 (±0.2) mm, with a median of 1.1 and a range of 0.6 mm to 1.5 mm. In 28% of the cases, the thickness was below 1.0 mm, and in 3% of the cases, it was thinner than 0.8 mm (Table 2).

## Discussion

This CBCT study evaluated the root and root canal configurations and root wall thickness of maxillary first premolars. A wide variation in the incidence of the numbers of roots, number of canals and configuration of the canals was found.

The distribution of the number of roots in the present study was similar to that reported in a number of studies in non-Asian populations (USA, Brazil, Poland, Spain, Jordan, Australia): 35% of the first maxillary premolars were reported to have one root, 66% two roots, and 3% three roots^3^. However, the numbers are different when Asian populations (South China, Singapore, Japan) were considered^3^. In a recent CBCT study from a Chinese population, one-rooted teeth were the most common (66%), whereas two-rooted teeth were found in only 33% of cases^17^. A recent review, which pooled data from 26 studies, reported that 41% of first maxillary premolars had one-rooted morphology and 57% had a two-rooted form^3^. The differences between these findings and those of the present study may be explained by the ethnic background of the studied populations^15^. Additional possible reasons for the differences are the method of study, technical characteristics of the method used (*i.e*., voxel size), and, above all, the definition of two separate roots and the definition of a canal^15^. In the present study, the most prevalent configuration of root canal system in first maxillary premolars was Vertucci Type IV, totaling 74% when all (400) teeth were considered. This number is quite similar to the number reported in a CBCT study in the Turkish population (77%)^16^, but it varied in other studies, all of which were performed using CBCT analysis: from 51 to 76%^12–14^.

When considering only one-rooted teeth, the most prevalent type was Vertucci Type II (48%), followed by Vertucci Type IV (31%), which is similar to that reported in the study in the Chinese population but differs from that reported in other CBCT studies^12–14^.

Overall, the anatomy of the first maxillary premolars is variable, and the reported difference can be explained in part by differences in the definition of a one-rooted *vs*. two-rooted teeth in the case of fused two roots. The resolution of the CBCT images and the analysis technique (multiple plane *vs*. axial plane analysis alone) may also have contributed to the variation in results.

**Figure 2:**
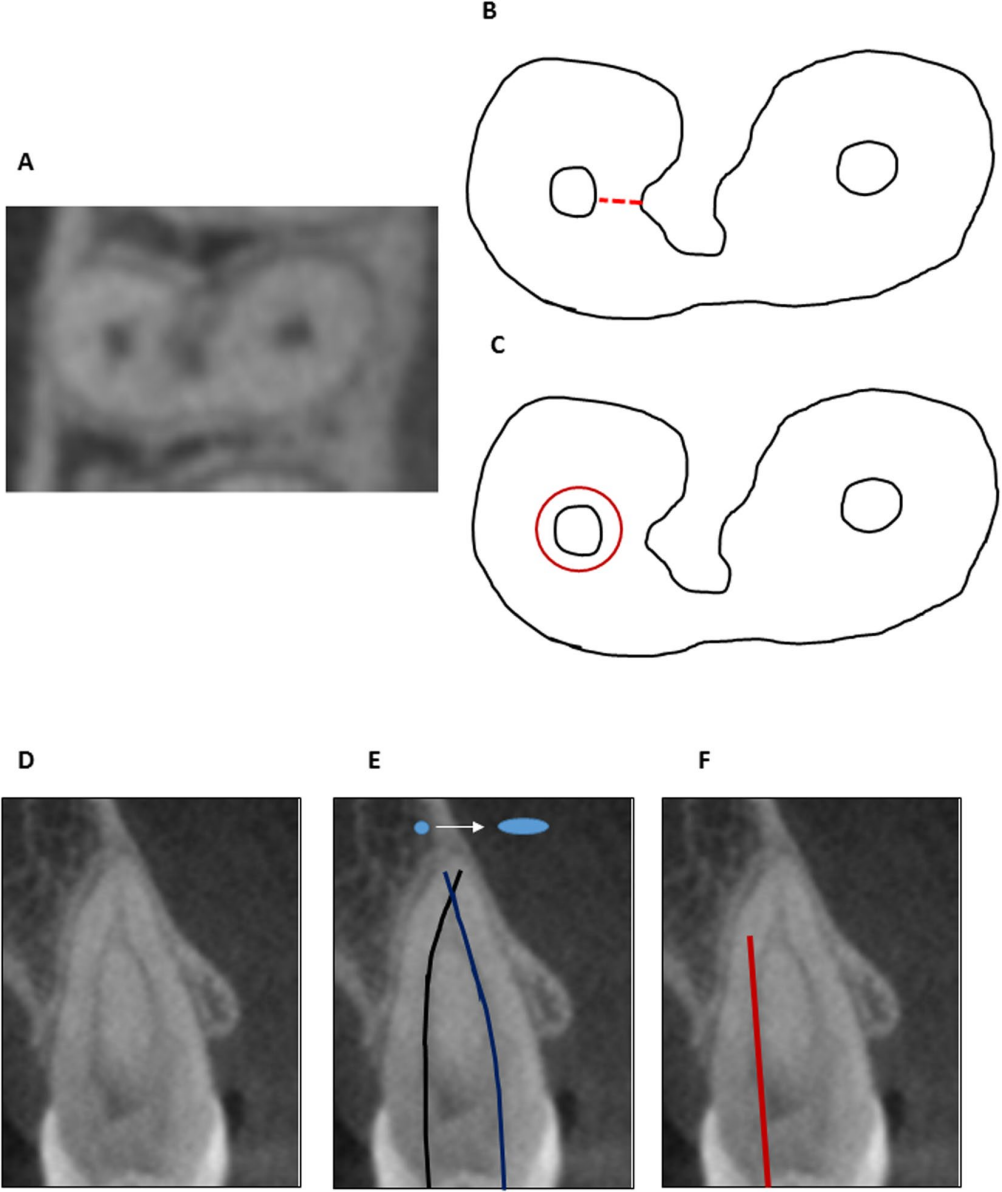
Potential pitfalls that cannot be recognized in two-dimensional periapical radiographs. (**A**) Axial section from CBCT of a two-rooted maxillary first premolar (fused roots) with a concavity in the buccal root facing the furcation. (**B**) Enlarged tracing of “A”. The red dotted line represents the wall thickness at the area of the concavity, which in this case was only 0.75 mm. (**C**) Enlarged tracing of “A”. Red circles represent preparation with a rotary file with a 1.2 mm diameter at D16. (**D**) Bucco-lingual section from CBCT of a single-rooted first maxillary premolar with a Vertucci Type II canal configuration. (**E)** Potential endodontic procedure pitfall: if thick rotary files are used (path marked with blue and black lines) to the measured working length, the apical foramen is likely to be zipped into an oval opening (blue) while destroying the apical constriction. (**F)** Potential restorative pitfall: post space preparation for a straight, long post (red line) to be placed in the bucco-lingually curved palatal canal may result in excessively thin remaining walls. Both pitfalls E and F could be avoided if the premolar was recognized as a single-rooted tooth, in which the Vertucci Type II canal configuration is common (48% in the present study).

One of the interesting results of the present study is the relatively high number of Vertucci Type II canal systems in single-rooted teeth (Table 2). This configuration may have a far-reaching clinical significance. When two canals of this type were present, they usually merged in a rather sharp angle (Fig. 2D), which may easily be overlooked if only conventional periapical radiographs are used, as such 2D radiographs do not demonstrate the bucco-palatinal dimension. This anatomical feature can be important in planning the mechanical shaping of canals in single-rooted premolars (Fig. 2E). It should be kept in mind that the two canals, in this type of canal configuration, are often *curved* in the bucco-palatal plane (Fig. 2D), which also cannot be seen/evaluated in a periapical radiograph. Additionally, when a post is to be placed in a tooth with such a curved canal configuration, its length and thickness should be limited to avoid perforation or excessive thinning of the root wall at the apical end of the post space (Fig. 2F).

A furcation groove in the buccal root was found in the present study in 58% of the two-rooted first maxillary premolars. This number is lower than that traditionally reported in the literature. The difference can be explained by two factors: first, the lower number of specimens in previous studies *vs*. the 400 teeth used in the present one, and second and most likely, the definition of two-rooted teeth. It is possible that in studies that used extracted teeth for microCT or for slicing and microscopy, two-rooted teeth with a prominent furcation divergence were chosen, whereas in the present study, the teeth with a more apically positioned furcation were also defined as being two rooted.

The mean root wall thickness at the most coronal part of the buccal root furcation groove (when present) was 1.1 (±0.3) mm. This value is slightly higher than that reported in the literature: 0.7–0.8 mm^8,18^. This difference can be explained by the methods: previous studies on this matter were performed using either microCT or physical slicing of the roots, and possible pre-selection of diverging two roots. The resolution of the chosen methods may also affect the results, as may the number of specimens in a study: 23–42 specimens in previous studies *vs*. 142 teeth with a groove in the current study. Additional contributing factors to the difference could be the canal diameter to dentin thickness ratio: large canal diameters and thus thinner root walls, may be found in younger populations.

The buccal root of first maxillary premolars is not usually recommended for post placement^19^. The present study supports this approach because the dentin thickness in the palatal groove area of the buccal roots was approximately 1 mm before instrumentation, but thinner walls were found in the present study in 39% of the teeth presented with a palatal groove in the buccal root. Furthermore, in 11% of these teeth, the thickness was less than 0.8 mm (Table 2). Clinical endodontics is not performed on an “average tooth” and the operator should be aware of the rather wide range of wall thickness and that the specific tooth to be treated may have a much thinner wall than the average.

Although the walls of the palatal canal at the CEJ level were rather thick, the mean thickness of the palatal root wall at 5 mm apical to the CEJ level, where post space preparations commonly reach, was 1.1 (±0.2) mm, and in 28% of the cases thinner than that. This finding is of clinical importance because the palatal root is commonly used for post space preparation and post placement.

Post space preparation may involve removing as much as 31% of root wall thickness^20^; thus, the procedure may leave residual root dentin thickness below the optimal level of 1 mm. The case may be even more extreme when post preparation in a palatal canal of a single-rooted tooth is considered. When a single-rooted tooth has a Vertucci Type II canal system, in which the canal is often curved in the bucco-lingual plane, preparing a post space may present a potential danger (Fig. 2E,F). It may be of clinical importance to know in advance that the palatal canal is curved when post preparation in this canal is planned. This cannot be estimated from conventional periapical radiographs.

These characteristics of single rooted first maxillary premolars, together with their narrow mesio-distal diameter compared to the bucco-lingual dimension, may explain the predisposition of the restored upper first premolar to vertical root fracture^11,21–23^.

Commonly used mechanized file systems, such as ProTaper Next X2 (Dentsply-Maillefer, Ballaigues, Switzerland), have diameters of 1.2 mm at D16 and 1.1 mm at D13^24^. Thus, canal preparation with such files will result at least with the above diameters. These diameters should be considered together with the mean diameter of the un-instrumented canal in the coronal area of first maxillary premolars, which is 0.54 mm^18^. Such a comparison may indicate that in the case of an instrument with a 1.2 mm diameter, at D16, reducing the thickness of the dentin wall by 0.33 mm at the coronal third of the canal may be expected (1.2 mm preparation minus 0.54 diameter of the un-instrumented canal divided by 2).

A canal wall that was initially 1.0 mm thick may thus be reduced to 0.67 mm, and when the original wall thickness is 0.8 mm to 0.47 mm. Consequently, the wall thickness after the endodontic instrumentation will be markedly below the commonly recommended level of 1.0 mm remaining wall thickness. Post preparation in these teeth may further reduce the thickness of the remaining dentin wall (Fig. 2C).

Taken together, the fact that the mean thickness of the root canal wall in critical areas of both buccal and palatal roots is approximately 1 mm before any instrumentation and may be thinner than that in many individual cases (Table 2, Fig. 2) may be of great clinical significance.

Becoming familiar with the wall thickness of first maxillary premolar roots, but especially, with the *wide range* of this parameter, should encourage clinicians to check the individual tooth to be treated not only for number and shape of canals but also for its *wall thickness* at the critical points mentioned in this study, which can only be done by using CBCT, preferably with limited FOV.

A limitation of the present study was that a more detailed data regarding the palatal groove (starting point and length) was not collected. It should be the focus of a future study. Additional large scale studies using similar concept may be required on other teeth in which the canal configuration and root wall thickness cannot be assessed from common periapical radiographs. This may include mandibular incisors and mesial roots of mandibular molars in which the above parameters may greatly affect the clinical outcome of the endodontic procedure.

The results of the present study suggest that when the patient has a previous CBCT that include the first maxillary premolars, one should not limit his attention to detecting roots and root canal configuration, but check also the axial plane view for the *thickness of the dentin wall* at the critical points, so as to apply a more conservative shaping approach in cases with thinner walls. Minimally invasive endodontic instrumentation^25^ and restorative methods may then be applied, as what can be termed “Personalized Endodontic Treatment”: Rather than following a standard protocol for instrumentation of the canals of first maxillary premolars, adapting it to the canal configuration and wall thickness of the specific individual tooth.

## Conclusions

1. The canals of single-rooted first maxillary premolars often have a Vertucci Type II configuration, with substantial curvatures in the bucco-palatal plane. This factor should be considered during both endodontic and the subsequent restorative treatment of these teeth.
2. Considering the limited thickness of the root dentin walls of first maxillary premolars, at critical points, minimally invasive endodontic shaping (using smaller instruments) and post preparations should be considered in these VRF-prone teeth.
3. Neither the configuration of the canal system nor the thickness of the walls can be evaluated from periapical radiographs alone, thus if the patient has a *previous* CBCT that includes the first maxillary premolars, it should be evaluated and analyzed before treating and restoring these challenging teeth.
